# Linkage Disequilibrium Estimation in Low Coverage High-Throughput Sequencing Data

**DOI:** 10.1534/genetics.118.300831

**Published:** 2018-03-26

**Authors:** Timothy P. Bilton, John C. McEwan, Shannon M. Clarke, Rudiger Brauning, Tracey C. van Stijn, Suzanne J. Rowe, Ken G. Dodds

**Affiliations:** AgResearch, Invermay Agricultural Centre, Mosgiel 9053, New Zealand

**Keywords:** genotyping-by-sequencing, linkage disequilibrium, maximum likelihood, allelic dropout, low coverage

## Abstract

High-throughput sequencing methods that multiplex a large number of individuals have provided a cost-effective approach for discovering genome-wide genetic variation in large populations. These sequencing methods are increasingly being utilized in population genetic studies across a diverse range of species. Two side-effects of these methods, however, are (1) sequencing errors and (2) heterozygous genotypes called as homozygous due to only one allele at a particular locus being sequenced, which occurs when the sequencing depth is insufficient. Both of these errors have a profound effect on the estimation of linkage disequilibrium (LD) and, if not taken into account, lead to inaccurate estimates. We developed a new likelihood method, GUS-LD, to estimate pairwise linkage disequilibrium using low coverage sequencing data that accounts for undercalled heterozygous genotypes and sequencing errors. Our findings show that accurate estimates were obtained using GUS-LD, whereas underestimation of LD results if no adjustment is made for the errors.

## Abstract (precis)

High-throughput sequencing methods provide a cost-effective approach for genotyping and are commonly used in population genetics studies. A drawback of these methods, however, is that sequencing and genotyping errors can arise...

LINKAGE disequilibrium (LD) is the term given to the nonrandom association of alleles located at different loci in a population. Quantifying the level of LD, or estimating the pairwise LD between all loci in a population, is of interest to many researchers as it has many important applications. For example, in association mapping studies, LD is used to identify candidate regions of the genome associated with a particular trait or disease, and can provide finer resolution in mapping compared to linkage-based studies (Devlin and Risch 1995; Jorde 1995; Xiong and Guo 1997; Mackay and Powell 2007). LD is affected by many genetic and evolutionary forces, such as recombination, admixture, migration, selection, and gene flow among others (Terwilliger *et al.* 1998; Ardlie *et al.* 2002; Gaut and Long 2003; Slatkin 2008). Consequently, LD patterns can be used to quantify genetic diversity and make inferences about the evolutionary history of natural populations (Nordborg and Tavaré 2002; Slatkin 2008; Zhu *et al.* 2015). In addition, the relationship between map distance and the level of LD can be used to estimate the effective population size (Sved 1971; Hill 1981; Hayes *et al.* 2003; Waples 2006; Sved *et al.* 2013).

Today, many species are being sequenced using high-throughput sequencing methods that multiplex a large number of individuals. Some of the most popular sequencing methods are whole genome sequencing, and reduced representation approaches such as genotyping-by-sequencing (Elshire *et al.* 2011), whole-exome sequencing (Hodges *et al.* 2007), and restriction-site associated DNA (Baird *et al.* 2008). These sequencing methods provide a low-cost approach to performing genome-wide genotyping and discovery of single nucleotide polymorphisms (SNPs) that does not require prior genomic information. As a result, they have been applied in a plethora of plant, aquaculture, and animal species, and have become the method of choice for many species, particularly for nonmodel organisms (Andrews *et al.* 2016; Kim *et al.* 2016; Chung *et al.* 2017; Li and Wang 2017; Robledo *et al.* 2017). Genetic data generated using high-throughput sequencing methods are increasingly being used to compute pairwise LD estimates (*e.g.*, Hohenlohe *et al.* 2012; Wang *et al.* 2013; Huang *et al.* 2014; Nimmakayala *et al.* 2014; Xu *et al.* 2014; Fè *et al.* 2015; Zhang *et al.* 2015; Covarrubias-Pazaran *et al.* 2016; Van Wyngaarden *et al.* 2016; Gur *et al.* 2017; Sieber *et al.* 2017; Faville *et al.* 2018).

A major disadvantage with high-throughput sequencing methods is that one or both of the alleles at a particular locus may be missed for a given individual if the sequencing depth is low. If neither allele is seen, a missing genotype results while if only one of the two parental alleles is seen (possibly multiple times), a heterozygous genotype may be called as homozygous (Dodds *et al.* 2015; Fragoso *et al.* 2016). The latter case is also known as allelic dropout, and is particularly problematic as genotype calls with this type of missingness behave like genotyping errors, which have a profound impact on the estimation of LD even when the error rates are low (Akey *et al.* 2001). An additional complication of sequencing data are the presence of sequencing errors, bases which have been miscalled, which also impact on estimation of genetic quantities such as recombination fractions (Bilton *et al.* 2018).

One way of removing genotyping errors resulting from low sequencing depth is to set genotype calls with an associated read depth below some threshold value to missing. However, such filtering results in fewer individuals and SNPs for a given sequencing cost (Dodds *et al.* 2015), and, for low coverage data, may result in insufficient data to undertake the analysis. LD is often estimated using haplotypes phased from genotype data via various software packages and algorithms such as BEAGLE (Browning and Browning 2007), fastPHASE (Scheet and Stephens 2006), MaCH (Li *et al.* 2010), and FILLIN (Swarts *et al.* 2014). However, all of these approaches require that the chromosomal order of the loci is known in order to infer haplotypes, which is not necessarily the case for reduced representation sequencing data, particularly if SNPs are called *de novo*. Furthermore, many species that are genotyped using sequencing methods are highly polymorphic and have low LD levels, where phasing in such species can be problematic (Bukowicki *et al.* 2016). A few alternative approaches for estimating LD from high-throughput sequencing data have been presented in the literature. Feder *et al.* (2012) proposed estimating pairwise LD using reads that cover both loci while estimating the allele frequencies using all the reads. This approach, however, is not applicable to short-read sequencing data (*e.g.*, genotyping-by-sequencing) where most of the reads do not cover both sites. Alternatively, it restricts the analysis to loci that are very close, which may not be that useful. Maruki and Lynch (2014) presented a likelihood method for estimating the disequilibrium coefficient in situations where there is a combination of reads that intersect both loci or only one of the two loci. Their method accounts for sequencing errors but requires that additional erroneous alleles are called in the alignment process, whereas most variant callers by default only allow for two alleles to be called at a SNP.

We present a new method for estimating pairwise LD using low coverage sequencing data, without requiring haplotype phasing, a known chromosomal order or filtering with regard to read depth. In essence, our method is based on the likelihood method by Hill (1974), which estimates LD using genotypic data in random mating populations, but is extended to account for errors resulting from undercalled heterozygotes and sequencing errors. Our method removes bias in LD estimation caused by these errors but results in more variable estimates at low depth. We also examine the effect genotyping errors from low read depths and sequencing errors have on the estimation of LD.

## Materials and Methods

### Estimation of pairwise LD

Let $A_{j}$ and $B_{j}$ denote the reference and alternate allele at locus *j*, respectively, and let $p_{A_{j}}$ and $p_{B_{j}}$ denote the allele frequency for the reference and alternate alleles at locus *j*, respectively. The LD coefficient is defined as (Lewontin and Kojima 1960):

$$D=p_{A_{1}A_{2}}-p_{A_{1}}p_{A_{2}},$$ (1)

where $p_{A_{1}A_{2}}$ is the probability of observing a haplotype containing the reference allele at both loci. Since probabilities are required to be non-negative, *D* must satisfy the constraints (Lewontin 1964):

$$\begin{array}{c} D\geq \max\left(-p_{A_{1}}p_{A_{2}},-\left(1-p_{A_{1}}\right)\left(1-p_{A_{2}}\right)\right)\\ D\leq \min\left(p_{A_{1}}\left(1-p_{A_{2}}\right),p_{A_{2}}\left(1-p_{A_{1}}\right)\right). \end{array}$$ (2)

We let $G_{ij}$ denote the true genotype for individual *i* at locus *j*, and $G_{i}={\left(G_{ij},G_{ik}\right)}^{T}$ denote the true joint genotype for individual *i* between locus *j* and *k*, where j≠k, i=1,…,n and *T* denotes the transpose. We let $AA_{j},$ $AB_{j},$ and $BB_{j}$ denote the reference homozygous genotype, heterozygous genotype, and alternate homozygous genotype at locus *j*, respectively. For two biallelic loci, the nine joint genotypes are ${\left(AA_{1},AA_{2}\right)}^{T},$ ${\left(AA_{1},AB_{2}\right)}^{T},$ ${\left(AA_{1},BB_{2}\right)}^{T},$ ${\left(AB_{1},AA_{2}\right)}^{T},$ ${\left(AB_{1},AB_{2}\right)}^{T},$ ${\left(AB_{1},BB_{2}\right)}^{T},$ ${\left(BB_{1},AA_{2}\right)}^{T},$ ${\left(BB_{1},AB_{2}\right)}^{T},$ and ${\left(BB_{1},BB_{2}\right)}^{T},$ which we denote by 1–9, respectively.

In sequencing data, the true genotypes are latent while the observed data consists of the number of reads for the reference and alternate alleles. We denote the number of reads for the reference allele for individual *i* at locus *j* by $Y_{ij},$ where $Y_{ij}$ is an integer value between 0 and the sequencing depth $d_{ij},$ which is the sum of reference and alternate allele counts at locus *j* in individual *i*. By the law of total probability,

$$P\left(Y_{i}\right)=\sum_{g=1}^{9}P\left(Y_{i}|G_{i}=g\right)P\left(G_{i}=g\right),$$ (3)

where $Y_{i}={\left(Y_{i1},Y_{i2}\right)}^{T}$. If the number of observed reads for the reference allele given the true genotype are independent between loci, Equation (3) simplifies to

$$P\left(Y_{i}\right)=\sum_{g=1}^{9}\left(\prod_{j=1}^{2}P\left(Y_{ij}|G_{ij}=g_{j}\right)\right)P\left(G_{i}=g\right).$$ (4)

where *g_j_* is either *AA_j_*, *AB_j_* or *BB_j_*. The expected true joint genotype probabilities, $P_{ig}$=$P\left(G_{i}=g\right),$ correspond to those given in Table 1 when the population is in Hardy-Weinberg equilibrium (Hill 1974).

**Table 1:** Joint genotype probabilities for two biallelic loci under the assumption of Hardy-Weinberg equilibrium

*g*	Locus 1	Locus 2	$P_{ig}$
1	$AA_{1}$	$AA_{2}$	${\left(p_{A_{1}}p_{A_{2}}+D\right)}^{2}$
2		$AB_{2}$	$2\left(p_{A_{1}}p_{A_{2}}+D\right)\left(p_{A_{1}}p_{B_{2}}-D\right)$
3		$BB_{2}$	${\left(p_{A_{1}}p_{B_{2}}-D\right)}^{2}$
4	$AB_{1}$	$AA_{2}$	$2\left(p_{A_{1}}p_{B_{2}}+D\right)\left(p_{B_{1}}p_{A_{2}}-D\right)$
5		$AB_{2}$	$2\left(p_{A_{1}}p_{A_{2}}+D\right)\left(p_{B_{1}}p_{B_{2}}+D\right)+2\left(p_{A_{1}}p_{B_{2}}+D\right)\left(p_{B_{1}}p_{A_{2}}-D\right)$
6		$BB_{2}$	$2\left(p_{A_{1}}p_{B_{2}}+D\right)\left(p_{B_{1}}p_{B_{2}}+D\right)$
7	$BB_{1}$	$AA_{2}$	${\left(p_{B_{1}}p_{A_{2}}+D\right)}^{2}$
8		$AB_{2}$	$2\left(p_{B_{1}}p_{A_{2}}+D\right)\left(p_{B_{1}}p_{B_{2}}-D\right)$
9		$BB_{2}$	${\left(p_{B_{1}}p_{B_{2}}-D\right)}^{2}$

The number of reads for the reference allele, $Y_{ij},$ can be considered as arising from a binomial sample of the two alleles found in the true genotype $G_{ij}.$ Suppose that the alleles are read at random, and that sequencing errors for a given read are independent between loci, the conditional probabilities of the number of reference alleles given the true genotype are:

$$\begin{array}{c} P\left(Y_{ij}=a|G_{ij}=AA_{j}\right)=\left(\begin{array}{c} d_{ij}\\ a \end{array}\right){\left(1-\varepsilon \right)}^{a}\varepsilon^{d_{ij}-a}\\ P\left(Y_{ij}=a|G_{ij}=AB_{j}\right)=\left(\begin{array}{c} d_{ij}\\ a \end{array}\right){\left(\frac{1}{2}\right)}^{d_{ij}}\\ P\left(Y_{ij}=a|G_{ij}=BB_{j}\right)=\left(\begin{array}{c} d_{ij}\\ a \end{array}\right)\varepsilon^{a}{\left(1-\varepsilon \right)}^{d_{ij}-a}, \end{array}$$ (5)

where *ε* is the sequencing error rate (Bilton *et al.* 2018). Assuming that individuals are independent (*e.g.*, unrelated), then the log-likelihood for the number of reference alleles is,

$$\ell \left(p_{A_{1}},p_{A_{2}},D,\varepsilon \right)=\sum_{i=1}^{n}\ln\;P\left(Y_{i}\right).$$ (6)

The maximum likelihood estimate of the disequilibrium coefficient, $\hat{D},$ using sequencing data are obtained by maximizing the likelihood in Equation (6) subject to the constraint of Equation (2). As no analytical solution exists, maximization of the likelihood is performed using numerical methods. The expectation of the maximum likelihood estimate is (Weir 1996),

$$E\left(\hat{D}\right)=\frac{2n-1}{2n}D,$$ (7)

resulting in a small bias, which is removed by multiplying $\hat{D}$ by 2n/(2n−1) subject to constraint (2), where *n* is taken as the number of individuals with a nonzero read depth at both loci.

Since the range of *D* depends on the allele frequencies, comparing levels of LD between markers can be difficult using the disequilibrium coefficient. Consequently, many alternative measures of LD have been proposed in the literature; see Hedrick (1987) and Devlin and Risch (1995) for a summary and comparison of these measures. In this article, we shall only consider two commonly used measures, $D^{\prime }$ (Lewontin 1964; Hedrick 1987) and $r^{2}$ (Hill and Robertson 1968). Although both $D^{\prime }$ and $r^{2}$ are measures of LD, they have different properties and are useful for different applications (see Mueller (2004)). The maximum likelihood estimates for both of these measures are computed using the functions ${\hat{D}}^{\prime }=\hat{D}/{\hat{D}}_{\max}$ and ${\hat{r}}^{2}={\hat{D}}^{2}/\left({\hat{p}}_{A_{1}}\left(1-{\hat{p}}_{A_{1}}\right){\hat{p}}_{A_{2}}\left(1-{\hat{p}}_{A_{2}}\right)\right),$ where

$${\hat{D}}_{\max}=\{\begin{array}{cc} \min\left({\hat{p}}_{A_{1}}{\hat{p}}_{A_{2}},\left(1-{\hat{p}}_{A_{1}}\right)\left(1-{\hat{p}}_{A_{2}}\right)\right) & \hat{D}<0\\ \min\left({\hat{p}}_{A_{1}}\left(1-{\hat{p}}_{A_{2}}\right),{\hat{p}}_{A_{2}}\left(1-{\hat{p}}_{A_{1}}\right)\right) & \hat{D}>0, \end{array}$$ (8)

and ${\hat{p}}_{A_{1}}$ and ${\hat{p}}_{A_{2}}$ are the maximum likelihood estimates of the reference allele frequencies at locus 1 and 2, respectively. We refer to the proposed methodology as genotyping uncertainty with sequencing data-linkage disequilibrium (GUS-LD, pronounced *guzzled*).

### Simulation

To examine the performance of GUS-LD, a simulation study was undertaken. Generation of simulated sequencing data proceeded as follows. For each individual, two haplotypes were sampled from the four possible haplotypes for preset values of $p_{A_{1}},$ $p_{A_{2}},$ and *D*, and were then converted to genotype calls. Simulation of sequencing data proceeded by first generating a read depth for each individual at each locus by simulating realizations from a Poisson distribution with mean $\mu_{k_{j}},$ where a range of read depths were used ($\mu_{k_{j}}=1,2,3,4,5,7.5,10,15$). At each locus within each individual, alleles were sampled from the genotype call with equal probability and replacement until a sample size corresponding to the read depth was obtained, with a sequencing error (*e.g.*, $A_{j}$ being called as $B_{j}$ and vice versa) simulated to occur with probability *ε*. In some cases, the simulated read depth was zero resulting in a missing genotype. The simulations were performed under various combinations of $p_{A_{1}},$ $p_{A_{2}},$ and *D* (see Table 2 for a list of combinations used) and a fixed sequencing error rate of 1% (ε=0.01).

**Table 2:** Combinations of parameters used in the simulations

Simulation	$p_{A_{1}}$	$p_{A_{2}}$	*D*
1	0.5	0.5	−0.15, 0, 0.05, 0.15, 0.25
2	0.5	0.75	−0.01, 0, 0.05, 0.1, 0.125
3	0.9	0.9	−0.01, 0.03, 0.06, 0.09

Two sets of simulations were performed. The first compares estimation of LD using simulated sequencing data between GUS-LD and the standard likelihood procedure of Hill (1974) that assumes accurate genotype calls. For each combination of parameters, 10,000 simulated datasets of 100 individuals were generated, where estimates of the bias and standard error (SE) of $\hat{D},$ ${\hat{D}}^{\prime },$ and ${\hat{r}}^{2}$ were computed for both methods. In the second set, the optimal sequencing depth for a given sequencing effort, defined as the number of reads which is the product of the number of individuals, the number of loci, and the mean read depth, is examined. For each combination of parameters, 10,000 datasets were simulated, where the number of individuals in the datasets were set such that an average sequencing effort of 600 reads was maintained. Estimates of the LD measures were obtained using GUS-LD and the standard approach where the mean square errors of $\hat{D},$ ${\hat{D}}^{\prime },$ and ${\hat{r}}^{2}$ were computed.

### Deer dataset

GUS-LD was also compared to the standard likelihood approach using a dataset consisting of 666 farmed deer and 38 of their sires. The dams were unrecorded red deer (*Cervus elaphus*) while the sires were predominantly Wapiti (also known as Elk; *Cervus canadensis*), but included some red deer. The animals were managed in accordance with the provisions of the New Zealand Animal Welfare Act 1999, and the Codes of Welfare developed under sections 68−79 of the Act. Tissue samples were collected in the form of ear tissue punches and DNA extracted according to Clarke *et al.* (2014). Genotyping was performed using the genotyping-by-sequencing method (Elshire *et al.* 2011) using the restriction enzyme *Pst*I and variations of the standard laboratory methodology as outlined in Dodds *et al.* (2015). The individuals were sequenced across eight lanes at AgResearch, Invermay, Animal Genomics laboratory on an Illumina HiSeq 2500 v4 chemistry yielding ∼1.34B reads (read length of 1× 100 bp) in total. SNP variants were called using UNEAK (Lu *et al.* 2013) as outlined in Dodds *et al.* (2015). For the LD analysis, a set of 38 SNPs that were determined to be close to the microsatellite TGLA94 (Marshall *et al.* 1998), had a minor allele frequency >0.05, and had <25% missing genotype calls were retained for analysis.

### Data availability

Scripts for generating the simulated sequencing data are provided in Supplemental Material, File S1. The deer dataset and an implementation of GUS-LD can be found at https://github.com/AgResearch/GUS-LD. Figures S1 and S2 in File S1 gives bias and SE of LD estimates for the second and third simulation scenarios. Figure S3 in File S1 gives the SE of the allele frequency estimates for all the simulations. Figures S4 and S5 in File S1 gives the mean square errors of LD estimates for the second and third simulation scenarios. Figure S6 in File S1 gives the mean read depth distribution for the SNPs used in the deer dataset and Figure S7 in File S1 gives the distribution of the sequencing error estimates for the deer analysis. Supplemental material available at Figshare: https://doi.org/10.25386/genetics.6007730.

## Results

### Simulation

**Figure 1:**
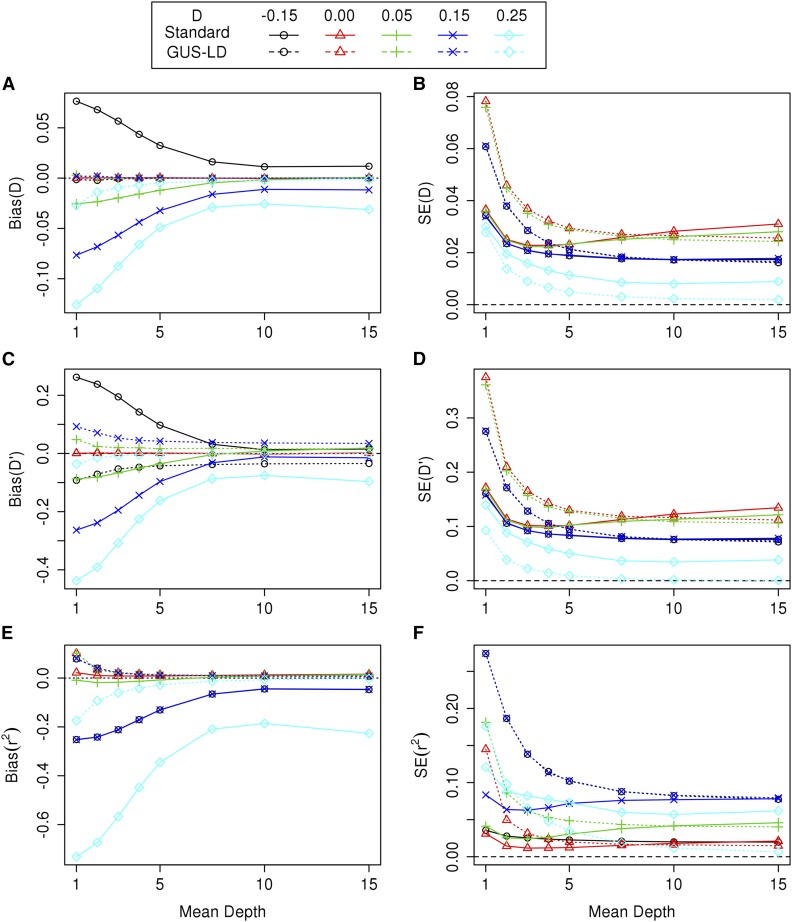
Bias of the LD estimates for *D* (A), $D^{\prime }$ (C), and $r^{2}$ (E), and SE of the LD estimates for *D* (B), $D^{\prime }$ (D), and $r^{2}$ (F) when $p_{A_{1}}=0.5,p_{A_{2}}=0.5,$ ε=0.01, and the true values of *D* were −0.05, 0, 0.05, 0.15, and 0.25. The dashed lines represents the estimates obtained using GUS-LD whereas the solid lines represents the estimates obtained using the standard likelihood approach. The upper and lower bounds for *D* are −0.25 and 0.25, respectively.

For the first set of simulations, the bias of the LD estimates for the various LD measures are given in Figure 1, for $p_{A_{1}}=0.5,p_{A_{2}}=0.5$ and for a range of values of *D*. When the average read depth was low, the estimates of *D* obtained using the standard likelihood procedure were biased toward zero, where the level of bias increased as the strength of LD increased. In contrast, the estimates computed using GUS-LD were relatively unbiased across the various read depths. Nevertheless, for the cases when *D* was close to, or on, its upper or lower bound [Equation (2)], $\hat{D}$ was biased, although the level of bias was much less for GUS-LD than for the standard likelihood procedure. These conclusions, in general, also applied to estimation of $D^{\prime }$ and $r^{2},$ although there was some bias in the estimates of $D^{\prime }$ even when the read depth was large and the true value of *D* was not near the upper or lower bound of its parameter space. This bias is due to poor sampling properties of $D^{\prime },$ and has been observed to occur in simulation studies for small sample sizes (Teare *et al.* 2002; Terwilliger *et al.* 2002). As the average read depth increased, the number of undercalled heterozygous genotypes in the datasets decreased, resulting in less bias for LD estimates obtained from the standard likelihood method. For mean depths >10, the estimates from the standard approach coincided with GUS-LD when the true LD was small or absent but were still biased when the true LD was large, which is due to the presence of sequencing errors.

Figure 1 also shows the SE of the estimates for the three LD measures computed using the two approaches. In general, the SE of the LD estimates computed under GUS-LD were larger compared with those obtained under the standard likelihood approach, with the difference decreasing as the average read depth increased. This increase in the SE for GUS-LD was expected as there is extra sampling variation introduced into the sequencing data, caused by not all alleles being observed. On the other hand, when the true value of *D* was close to, or on, the lower or upper bound of its parameter space [Equation (2)], GUS-LD tended to yield smaller SE than the standard approach.

The bias and SE of the LD estimates for alternative combinations of allele frequencies are given in Figure S1 ($p_{A_{1}}=0.5$ and $p_{A_{2}}=0.75$) and Figure S2 ($p_{A_{1}}=p_{A_{2}}=0.9$) in File S1. The results from these simulations were mostly in agreement with those when $p_{A_{1}}=0.5\text{ and }p_{A_{2}}=0.5.$ The SE for the allele frequency estimates from GUS-LD and the standard approach for all three sets of parameter values are given in Figure S3 in File S1. Overall, the SE of the allele frequency estimates were fairly similar between the two methods.

**Figure 2:**
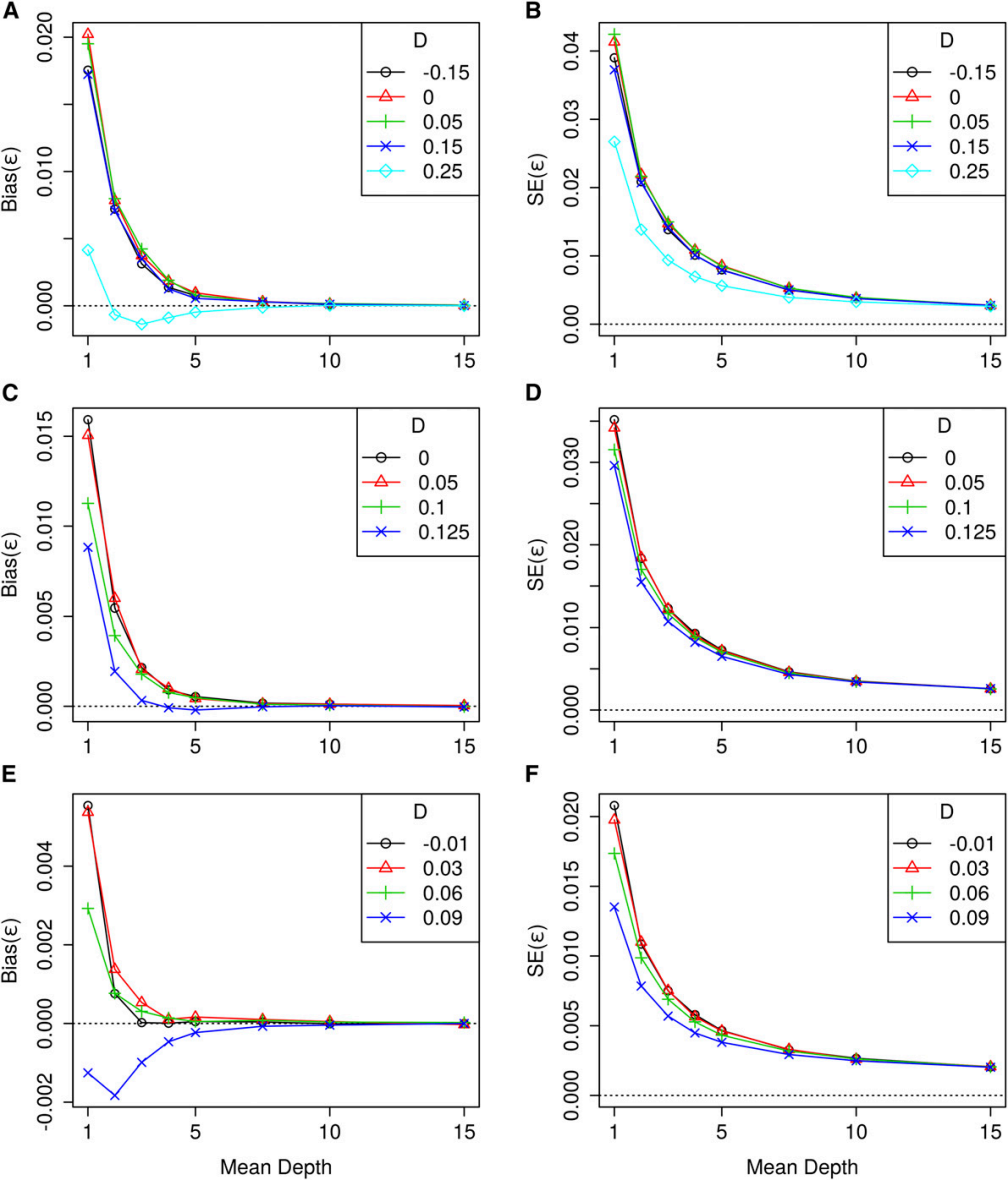
Bias of the sequencing error estimates, $\hat{\varepsilon },$ from GUS-LD for simulation 1 (A), simulation 2 (C) and simulation 3 (E), and SE of the sequencing error estimates, $\hat{\varepsilon },$ from GUS-LD for simulation 1 (B), simulation 2 (D), and simulation 3 (F), where the parameters used for each simulation are given in Table 2.

The bias and SE of the sequencing error estimates from GUS-LD for the first set of simulations is given in Figure 2. At high mean depths, these estimates were unbiased across all the different combinations of parameter values, whereas for low mean read depths the estimates were generally biased upwards, with the bias increasing as the mean depth decreased. The SE of the sequencing error estimates were also smallest at higher mean depths, and increased as the mean depth decreased.

**Figure 3:**
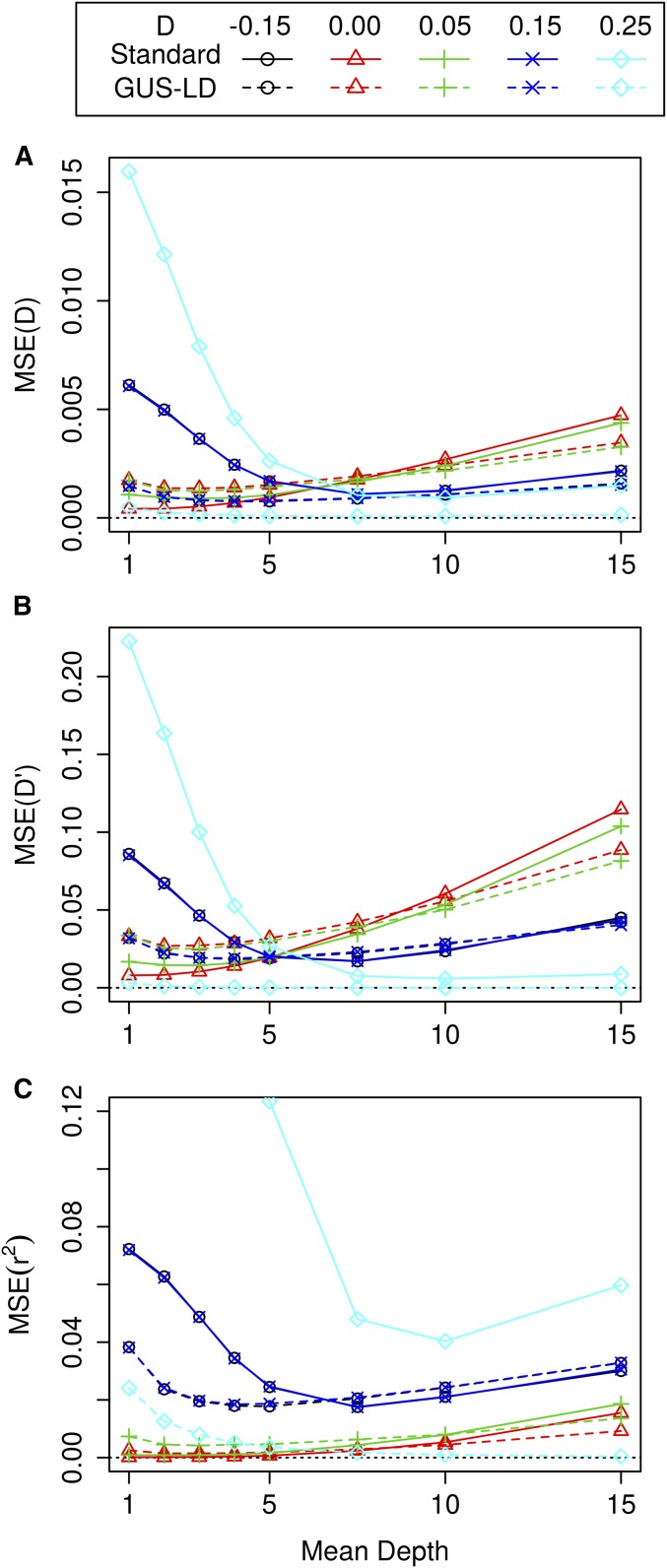
MSE of the LD estimates for *D* (A), $D^{\prime }$ (B), and $r^{2}$ (C) for a fixed average sequencing effort of 600 reads when $p_{A_{1}}=0.5,p_{A_{2}}=0.5,$ ε=0.01, and the true values of *D* were −0.05, 0, 0.05, 0.15, and 0.25. The upper and lower bound for *D* are −0.25 and 0.25, respectively. The dashed lines represents MSE for GUS-LD, whereas the solid lines represents MSE for the standard likelihood approach. The number of individuals in the simulated data sets were 300, 150, 100, 75, 60, 40, 30, and 20 at mean read depths of 1, 2, 3, 4, 5, 7.5, 10, and 15, respectively.

For the second set of simulations, the mean square error (MSE) of the LD estimates for the various pairwise LD measures are given in Figure 3, where the sequencing effort was fixed at 600 reads, $p_{A_{1}}=p_{A_{2}}=0.5,$ ε=0.01 and a range of values of *D* were used. The MSE for GUS-LD was lower than the standard approach when the true LD was large or near its maximum value. Compared to GUS-LD, the standard approach gave lower MSE at low depths when the true LD was small, which was due to the LD estimates having a small bias and smaller SE compared to GUS-LD. On the other hand, the presence of sequencing errors results in the standard approach having higher MSE at high depths compared to GUS-LD. The MSE for GUS-LD was smallest between mean depths of 2 and 5, where the actual depth at which the minimum occurred depended on the true value of *D* and the LD measure. The MSE is larger at higher read depths for GUS-LD as the increase in variability from having fewer individuals in the data sets was larger than the decrease in variability from having high read depths. There was one exception to this trend that occurred when the true value of *D* was equal to its upper bound (D=0.25) for all the LD measures. In this case, the MSE was largest at smaller mean read depths and decreased as the mean read depth increased. This is due to the fact that there is no variation or bias when the genotypes are accurate for values of *D* that are on their upper or lower bound, but there is variation when there is uncertainty in the genotype calls associated with low read depths.

The MSE of the LD estimates for alternative combinations of allele frequencies when the sequencing effort was fixed are given in Figure S4 ($p_{A_{1}}=0.5$ and $p_{A_{2}}=0.75$) and Figure S5 ($p_{A_{1}}=p_{A_{2}}=0.9$)in File S1. The results from these simulations were very similar to the case when $p_{A_{1}}=p_{A_{2}}=0.5,$ although there were some differences. For example, the MSE across all the mean depths for *D* was larger as the true value of *D* increased when $p_{A_{1}}=p_{A_{2}}=0.9,$ whereas the reverse was true when $p_{A_{1}}=p_{A_{2}}=0.5,$ and when $p_{A_{1}}=0.5$ and $p_{A_{2}}=0.75.$ Also, for $p_{A_{1}}=0.5$ and $p_{A_{2}}=0.75,$ the MSE for the LD measure $r^{2}$ did not decrease as the read depth increased when the true value of *D* was on its upper boundary (D=0.125), as for the other parameter combinations. This was due to unequal allele frequencies meaning that the estimates of $r^{2}$ were not near its upper bound of 1. These differences were due to the complex sampling properties of the various LD measures. Nevertheless, the optimal sequencing depth was mostly between 2 and 5 across all scenarios and LD measures.

### Deer dataset

**Figure 4:**
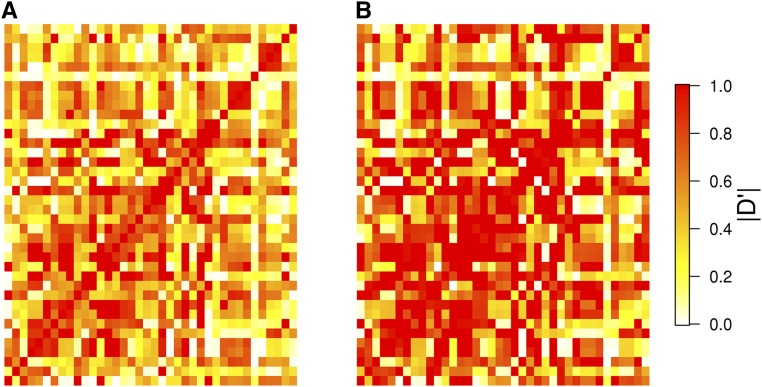
Heatmaps of the absolute value of the pairwise estimates for $D^{\prime }$ between all 38 SNPs in the deer dataset using (A) the standard likelihood approach, which does not account for undercalled heterozygous genotypes or sequencing errors, and (B) GUS-LD.

**Figure 5:**
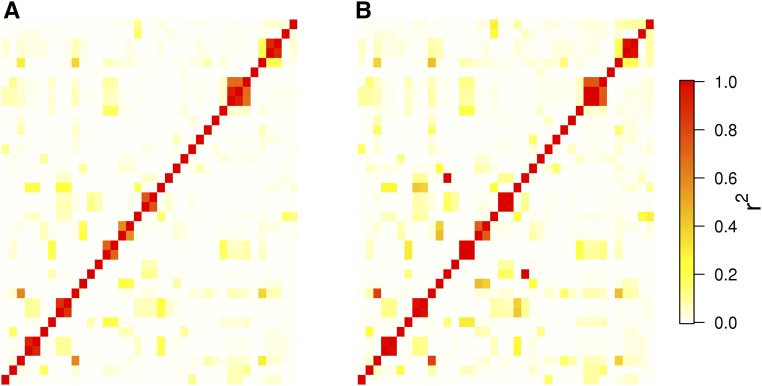
Heatmaps of the pairwise estimates for $r^{2}$ between all 38 SNPs in the deer dataset using (A) the standard likelihood approach, which does not account for undercalled heterozygous genotypes or sequencing errors, and (B) GUS-LD.

The LD estimates between all pairs among a set of 38 SNPs are given in Figure 4 for the absolute value of $D^{\prime }$ and Figure 5 for $r^{2}.$ For the former LD measure, a number of pairwise estimates computed using GUS-LD were larger compared to the estimates obtained from the standard likelihood approach, which is seen by the greater intensity of red across the heatmap in Figure 4B compared to Figure 4A. Similarly, there were some pairwise estimates of $r^{2}$ that were larger under GUS-LD (Figure 5B) compared to the standard likelihood approach (Figure 5A), which is seen by the fact that some of the yellow squares in Figure 5A appear more orange in Figure 5B. The average value of all the pairwise estimates for the two LD measures was larger under GUS-LD than the standard likelihood approach (Table 3). Compared to the simulation results, the difference in the LD estimates between the two approaches was not particularly large. This was due to a number of SNPs having high mean read depths (Figure S6 in File S1). Nevertheless, the *P*-values from a Wilcoxon signed-rank test comparing the mean LD estimated from GUS-LD and the standard approach were very small (Table 3), giving strong evidence that the mean estimated level of LD from GUS-LD was significantly larger than from the standard approach. The distribution of the sequencing error estimates obtained from GUS-LD for all SNP pairs is given in Figure S7 in File S1, where the mean estimate was 0.14%.

**Table 3:** Average LD estimate across all pairs of SNPs for the deer dataset

LD Measure	Standard	GUS-LD	*P*-value*^a^*
$\left|D^{\prime }\right|$	0.48	0.62	$<10^{-6}$
$r^{2}$	0.028	0.040	$<10^{-6}$

a *P*-value from a Wilcoxon signed-rank test comparing the mean level of LD estimated from the standard approach and GUS-LD. The test was performed in the programming language R (R Core Team 2017) using the wilcox.test function (paired = TRUE).

## Discussion

The introduction of high-throughput sequencing methods that multiplex a large number of individuals is driving forward research into many species, particularly nonmodel species, and is increasingly being utilized by many researchers. However, analyzing sequencing data using existing analytical tools and methods may, in some cases, be impractical or lead to erroneous results due to the added complexity and nuances of the data compared to other genetic data types. Consequently, the development of new methodological tools for analyzing sequencing data are needed, although the progress of such tools has been slow compared to the sequencing technology (Gardner *et al.* 2014).

Our simulation results have demonstrated that genotyping errors associated with undercalled heterozygotes (*e.g.*, allelic dropout), and miscalled bases leads to underestimation of LD when these errors are not taken into account. This is important, as biased estimates of LD can have a profound effect on downstream analyses. For example, in case-control association studies, it has been shown using simulations that the presence of genotyping errors leads to reduced power in detecting an association between a locus and phenotype (Gordon and Ott 2001; Gordon *et al.* 2002). Russell and Fewster (2009) have also shown via simulations that allelic dropout results in positively biased estimates of effective population size when calculated using LD information. This problem is exacerbated for low coverage data as the rate of genotyping errors is much higher than those used in these simulations studies. We have developed a new method, called GUS-LD, that accounts for errors associated with undercalled heterozygotes and miscalled bases in the estimation of LD. Our results show that GUS-LD was able to greatly reduce bias in LD estimates at low sequencing depth, although the variability of these estimates were larger compared to the standard approach at low depths, which reflects the additional variation introduced into the data by uncertainty over whether both alleles or only one allele were seen. This additional variation will affect downstream analyses such that there will be less power to detect causal variates in association studies, more variable estimates of effective population size and less precision in assessing genome quality. However, this can be counteracted by sampling more individuals, since this can be more efficient than sampling fewer individuals at high depth as suggested by our simulations results and by Maruki and Lynch (2014). The simulations also show that GUS-LD was able to reduce bias in LD estimates caused by sequencing errors, especially at high depths when the true LD was moderate to large.

The sequencing error parameter, *ε*, in GUS-LD is specified in terms of a miscalled base for a given read, which differs from the tradition specification that is in terms of a miscalled allele in a genotype call. As a consequence, GUS-LD estimates the sequencing error rate from information provided by the allele counts for the reference and alternate alleles. In addition, a smaller sequencing error rate under the alternative specification can affect more genotypes calls than under the traditional specification for the same value of *ε*, especially if there are many reads associated with each genotype call. This means that the estimate of *ε* from GUS-LD is likely to differ from sequencing errors rates generally quoted in the literature. For the deer data set, the mean sequencing error rate for a given read was estimated at ∼0.14%, which is of similar magnitude to the rate estimated by Bilton *et al.* (2018) in a linkage context for genotyping-by-sequencing data. Simulation results suggest that GUS-LD accurately estimates the sequencing error rate at high depths, but the estimates become biased as the mean depth decreases. This bias is likely due to the inability to distinguish between sequencing errors and true reads at very low depths. Nevertheless, GUS-LD still provided accurate LD estimates, even when the sequencing error estimates themselves were biased.

With low coverage sequencing data, there are issues with estimating LD when the true parameter value lies near or on the upper or lower bound of its parameter space [Equation (2)]. Specifically, the bias in the LD estimates increases as *D* approaches its upper or lower bound. This is even the case for GUS-LD, which adjusts for genotyping errors associated with low read depths, although the bias is significantly less than the standard likelihood approach. This bias is caused by sampling variation resulting in the maximum of the likelihood in Equation (6) lying outside the parameter space of *D*, whereas maximization is performed with respect to the constraint of Equation (2). When genotype calls are accurate and without error, this bias, in estimating *D* when its true value is near its upper or lower bound, is absent.

There are many potential applications of using pairwise LD estimates from GUS-LD. For example, they could be used for quantifying the extent of LD decay in populations relative to physical distance from an assembly or genetic distance computed from a linkage analysis. This should prove a popular application since there are numerous studies already using sequencing data for this purpose in a number of species (*e.g.*, Huang *et al.* 2014; Nimmakayala *et al.* 2014; Fè *et al.* 2015; Gur *et al.* 2017; Sieber *et al.* 2017), including one by Faville *et al.* (2018), which utilized GUS-LD. LD estimates from GUS-LD can also be used in conjunction with the method of Sved (1971) to estimate historic effective population size, or the method of Waples (2006) to estimate contemporary effective population size. Another application is assessing the quality of an assembly (*e.g.*, Pernaci *et al.* 2014) or ordering scaffolds, such as in the Locus Ordering by Dis-Equilibrium procedure (Khatkar *et al.* 2010). This application of LD is perhaps less well known but is particularly useful for sequencing data, since assemblies are often fragmented or not existent, and has already been used in a study by Tennessen *et al.* (2017). One powerful application is combining LD estimates from GUS-LD with the software package LDna (Kemppainen *et al.* 2015) to explore genome-wide LD and investigate the evolutionary forces acting on a population. The advantage of combining these two approaches is that no reference genome is required, meaning that it is applicable to any species and so will prove valuable for nonmodel species.

For the methodology developed in this paper, a number of assumptions have been made. First, genotype calls observed in the sequencing data are assumed to be conditionally independent between loci given the true genotype call. This assumption is reasonable provided that loci are not located on the same sequencing read across individuals. Estimation of LD is unaffected by the presence of genotyping errors resulting from low read depth when the loci are located on the same read as the true underlying haplotypes in the individuals are preserved. Depending on their settings, many variant callers allow for multiple SNPs to be called on the same sequencing read. However, it is more practical to only retain a single SNP from a given read as the loss of information is minimal and is outweighed by the reduced computational time. Other assumptions include that missing genotypes resulting from read depths of zero occur randomly, and that the alleles of the true genotypes are sampled randomly in the sequencing process. If the latter assumption does not hold, one allele will be sampled more frequently than the other (*e.g.*, preferential sampling). In this case, the proportion of heterozygotes seen as homozygotes will be larger than expected under the model, which would result in some bias in the LD estimates at low sequencing depth. If additional information is available, then the probabilities in Equation (5) can be adjusted to reflect alternative sampling models. Lastly, it is assumed that sequencing errors occur independently between reads. In reality, this assumption may not hold, although it has been found to be reasonable in some scenarios (Bilton *et al.* 2018).

The main contributions of this paper are twofold. First, we have demonstrated that there can be significant bias in LD estimates from sequencing data when the read depth is low and the associated errors are not taken into account. This highlights the need for practitioners to either remove these errors by filtering or adjust their methodology to account for these errors. This is particularly important as some LD analyses give no explicit mention of a minimum cut-off with respect to read depth being used. Second, we have proposed GUS-LD as a new method to estimate LD using low-coverage sequencing data. GUS-LD will prove valuable to researchers seeking to undertake population studies when cost constraints prohibit the production of high-coverage sequencing data or other types of genetic data. In fact, our simulation results suggest that it is more cost-efficient to use low coverage data, as it allows more individuals to be sequenced for the same cost and results in smaller mean square errors for the LD estimates. From our results, the optimal sequencing depth was between 2 and 5, which was similar to the optimal read depth observed by Dodds *et al.* (2015) in the context of relatedness estimation. GUS-LD also allows LD estimation using loci with a mixture of high and low mean read depths, which is particularly useful as the sequencing depth typically varies substantially between SNPs.
